# A proteomics approach to decipher the molecular nature of planarian stem cells

**DOI:** 10.1186/1471-2164-12-133

**Published:** 2011-02-28

**Authors:** Enrique Fernández-Taboada, Gustavo Rodríguez-Esteban, Emili Saló, Josep F Abril

**Affiliations:** 1 Departament de Genètica and Institute of Biomedicine (IBUB), Universitat de Barcelona, Av. Diagonal 645, 08028, Barcelona, Catalonia, Spain

## Abstract

**Background:**

In recent years, planaria have emerged as an important model system for research into stem cells and regeneration. Attention is focused on their unique stem cells, the neoblasts, which can differentiate into any cell type present in the adult organism. Sequencing of the *Schmidtea mediterranea *genome and some expressed sequence tag projects have generated extensive data on the genetic profile of these cells. However, little information is available on their protein dynamics.

**Results:**

We developed a proteomic strategy to identify neoblast-specific proteins. Here we describe the method and discuss the results in comparison to the genomic high-throughput analyses carried out in planaria and to proteomic studies using other stem cell systems. We also show functional data for some of the candidate genes selected in our proteomic approach.

**Conclusions:**

We have developed an accurate and reliable mass-spectra-based proteomics approach to complement previous genomic studies and to further achieve a more accurate understanding and description of the molecular and cellular processes related to the neoblasts.

## Background

As we move further into the post-genomic era it becomes increasingly clear that DNA sequence data alone is insufficient to explain complex cellular and molecular processes. Although the enormous volume of data generated by genome sequencing projects, expressed sequence tags (ESTs), and cDNA analyses has improved our understanding of many processes, they often fail to reflect the influence of posttranscriptional modifications and protein interactions or offer a true reflection of protein levels or activity. Consequently, the role of specific proteins is relatively difficult to determine with confidence on the basis of mRNA expression or genomic data alone [1,2].

Proteomic approaches offer a more realistic description of protein function and its influence on cell dynamics. Although comparative analysis of phenotypically different biological samples, such as in diseased versus healthy tissue [3], remains a challenge, those studies raise the possibility of identifying the protein "signatures" that underlie key biological phenomena [4]. Furthermore, the use of bioinformatics to integrate data obtained using genomic and proteomic techniques could help to bypass the limitations of each approach and achieve a more comprehensive view of the information flow within cells.

**Figure 1:**
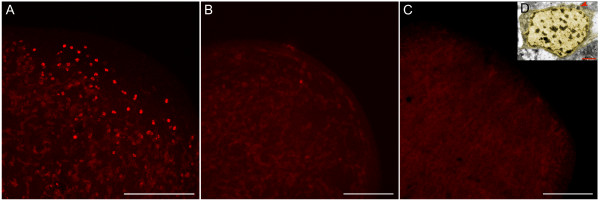
**Neoblast depletion by irradiation and image of a neoblast shown by electron microscopy**. Immunostaining with anti-phosphorylated histone H3 (αH3P), labelling mitotic neoblasts in 3-day head-regenerating organisms: A, control; B, 75 Gy irradiated 3 days after irradiation; and C, 75Gy irradiated 14 days after irradiation. Whereas a high number of proliferating cells appear in control animals next to the blastema and some mitotic cells still remain 3 days after irradiation, no divisions are detected after 14 days, showing that neoblasts are completely eliminated at that time. D, Electron microscopy image of a neoblast cell. Cytoplasm (dim yellow) and nucleus (yellow) are highlighted for clarity. The red arrow indicates a chromatoid body. Scale bars: A-C = 0.5 mm, D = 3 μm.

Planarians, an emerging model system for the investigation of stem cell and regenerative biology, [5-7], have a unique population of stem cells called neoblasts (see Figure 1), which can give rise to all of the differentiated cell types present in the adult organism during regeneration or normal homeostasis [8,9]. Albeit a great deal is now known about the biology of these cells, most molecular data have come from cDNA and genomic analyses. The neoblasts are particularly suited to proteomic approaches, however, as they contain chromatoid bodies (CB) that are progressively lost during differentiation [10-12] and can be employed as a marker for undifferentiated cells. The CB complexes are mainly formed by proteins and latent mRNA molecules, which can distort the levels of gene expression in transcriptional analyses of neoblasts samples. Moreover, since the neoblasts are the only dividing cells in the planaria [5], they can be easily depleted by irradiation [13]. Thus, these unique characteristics make planarians an ideal system in which to explore the use of proteomics to analyze the biology of processes such cell differentiation, stem cell behavior, homeostasis and an array of other events. As a first step in the development of such an approach, here we describe the methodological establishment and validation of a proteomic analysis of the planarian neoblast.

## Results

### Establishment of the planarian proteomic approach

**Figure 2:**
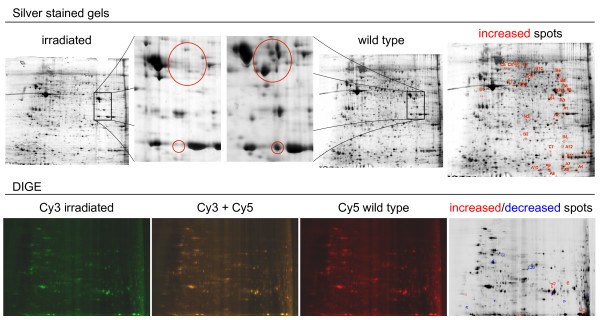
**Two-dimensional gels used for the selection of differential spots**. The proteomic approach shown compares the protein profile of a sample containing neoblast cells with one in which these cells have been depleted by irradiation. Upper panels show a comparison between two silver-stained 2D gels of a whole proteome from wild type and irradiated animals. Spots not present in the proteome of irradiated planarians are shown and lettered in red. These spots were selected and analyzed by mass spectrometry. Bottom panels show DIGE comparison of irradiated and wild type planarian proteomes. Spots that increase or decrease in the irradiated planarian proteome are shown in red and blue, respectively. These spots were included in the mass spectrometry analyses.

Different methods were tested to achieve a consistent and reproducible pattern on two-dimensional (2D) gels. To optimize sample preparation, proteins were extracted from dissociated cells or from whole animals. The yield from dissociated cells was insufficient to establish an efficient 2D procedure. Furthermore, the reproducibility of the 2D gel pattern was poor (data not shown). Prior to extraction from whole animals, a short treatment with 2% cysteine chloride in planarian water was used to eliminate mucous production, which is known to interfere with molecular techniques [14]. Based on our tests and previous work by Collet and Baguñà [15], we established a consistent method for 2D analysis from planarian samples (Figure 2 and Additional File 1). The different lysis buffers and sample cleaning procedures tested are shown in Table 1. Between 50 and 1000 μg of total planarian proteins were loaded on 2D gels to establish the best sample quantity in terms of spot definition. From 100 to 500 μg the spot resolution was acceptable. We selected the 500 μg as the optimal amount of protein to load onto 2D gels to achieve the maximum number of spots. A minimum of 100 μg was necessary for spot detection. Different immobilized pH gradient strips were used and the second-dimension protocol was modified to avoid streaking problems (Table 1). All these variables were tested on 12-cm 2D gels and scaled up to 24-cm gels for subsequent procedures.

**Table 1:** Variables taken into account for the establishment of the planarian proteomic protocol using 2D gels.

Samples:	Whole planarian extracts, dissociated cell extracts, dissociated cell and sub-fractionated extracts.
**Extraction Buffers:**	SDS, urea/thiourea.

**Processing Sample:**	(Precipitation procedure) Amersham 2D clean up kit, acetone, TCA-acetone.

**Isoelectric Focusing** **(1st Dimension):**	(Immobiline Dry strip gels 24 cm) Linear pH 4-7, Linear pH 7-11, Non-linear pH 3-11.

**Other Modifications:**	Trypsin inhibitors, general protease inhibitors, sonication.

All the different variables affecting protein sample production and 2D gel electrophoresis are listed on this table.

### Proteomic data

In order to identify proteins specifically expressed in neoblasts, we compared 2D patterns of two samples: wild type (WT) versus irradiated animals (IA). This method has been extensively used to study the effects of neoblast depletion [8,13]. Extractions were done 14 days after irradiation, when animals remained viable but cell proliferation was absent (Figure 1). Once the protocol was set up and the spot patterns were reproducible (Figure 2 and Additional File 2), the spots were compared and selected. Although spot labelling by silver staining and DIGE was consistent in each case, we did not succeed in obtaining a uniform pattern with the two techniques. Follow-up analysis was therefore done separately. With the aim of establishing the real potential of the silver-staining technique, only clear and conserved qualitative comparison based on silver staining was considered (present in WT sample and not present in irradiated sample). Image master 2D™software (from Amersham Biosciences) was used to analyze the scanned gels. However, the potential bottleneck of this proteomic approach is the image analysis. Many authors have highlighted the difficulties in obtaining good replicates [16], and this has now been partially overcome with the use of DIGE. Whereas our silver-staining results showed remarkable pattern conservation within replicates (Additional File 2), the numbers after spot image analysis showed some variability. In order to improve signal specificity we used two types of gels, one loaded with 100 μg and another with 500 μg of sample protein. The differences between irradiated and non-irradiated samples that were conserved in both sample loads and also had three surrounding reference spots in both experimental conditions were selected after reviewing the correspondence in Ip and Mw (Figure 2). These restrictions reduced the number of selected spots substantially, but ensured a high degree of confidence in the differences selected, providing a better platform for validation of the technique. For DIGE staining, the standard protocol was followed without modifications and the analysis software was used with the default parameters. Only clear and conserved quantitative changes (>2-fold changes) were selected, drastically reducing the number of final candidate spots (Figure 2). A total of 26 and 58 spots were selected for silver and DIGE staining, respectively (Table 2).

**Table 2:** Spot counts for the 2D gels.

	Semi-Automatic Procedure		Final Selected Spots
		
	Irradiated	Wild type	
100-SIL	1182 ± 43.13	901 ± 77.07	26
	
500-SIL	1931 ± 92.63	1413 ± 81.31	

500-DIGE	2445		58

Summarized data are shown for the 2D gel analyses. Image master 2DTM software (from Amersham Biosciences) was used to analyze the scanned gels. SIL, Silver staining; 100-SIL, 100 μg of total protein extract loaded on the gel; 500-SIL, 500 μg of total protein extract loaded on the gel; DIGE, differential in gel electrophoresis.

### Computational analyses

**Figure 3:**
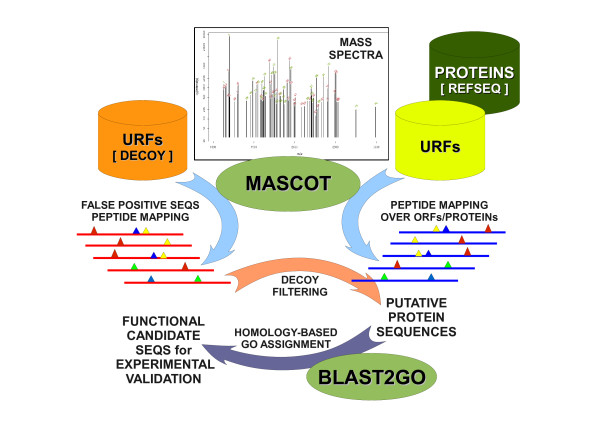
**Computational screening of protein candidates**. Spectra fingerprints were analyzed by MASCOT, comparing the experimental peaks against those obtained *in silico *from sequence databases. URFs were derived from planarian genome traces. Small triangles correspond to peptides found by MASCOT, mapped on candidate protein sequences for both databases, RefSeqs and URFs. Due to the size of the URFs database, a decoy approach was taken to select significant protein sequences. Putative protein sequences were ranked prior to experimental validation, taking into account MASCOT scores, number of peptide hits per sequence, decoy score, as well as functional assignment by BLAST2GO.

MASCOT [17] was tested against different open reading frame (ORF) datasets derived from NCBI-nr/RefSeq [18,19], *Schmidtea mediterranea *ESTs [20], the contigs for the planarian genome WUSTL assembly version 3.1 [21], and *S. mediterranea *whole-genome shotgun reads (traces). Of those datasets only NCBI-nr and traces are discussed here; the former is routinely used on this kind of analyses, while the latter yielded the largest number of peptide assignments (unpublished results). MASCOT assigned 20,107 peptides to spectra for NCBI-nr, which mapped to 602 protein sequences. Sequences from traces contained in the "forward" database were reversed to produce a "decoy" database containing sequences of the same length and composition but a different distribution of trypsin targets to those from the "forward"; Figure 3 illustrates the whole process. MASCOT returned 50 hits per search on each trace database, both for "forward" and "decoy". This resulted in 100 hits per search, for a total MS-fingerprint of 83 different spots.

**Figure 4:**
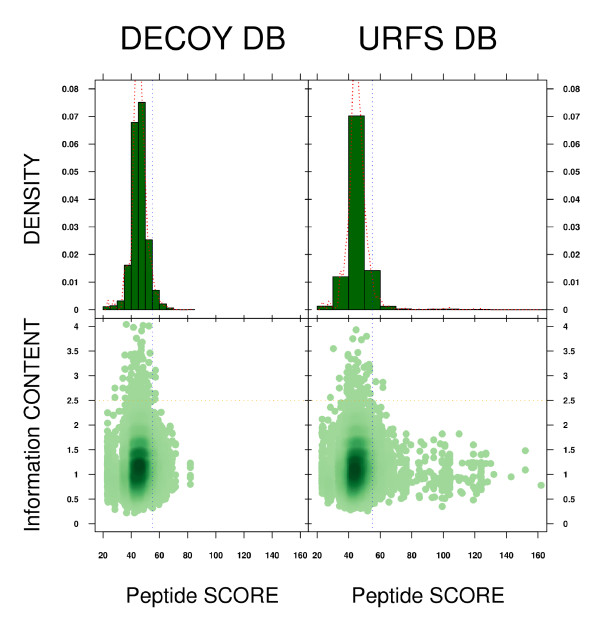
**Selection of candidate peptides by decoy score threshold**. Upper panels: histograms showing the distribution of the peptide scores (the maximum score was chosen when a peptide was mapped more than once to different open reading frames). Lower panels: scatter-plots comparing those peptide scores with the information content, in bits. Above a bit score of 2.5 (orange line), the peptide sequences can be considered of low complexity or repetitive. Decoy score threshold is depicted on all the panels as a vertical blue line, set at a score of 55 for our data.

MASCOT predicted a total of 44,712 and 36,956 peptides for the forward and decoy databases, respectively, and these were mapped to 8300 unique ORFs (URFs), corresponding to 23,376 and 26,741 unique peptide sequences. When the same peptide was mapped on two or more URFs, the highest score was retrieved. Figure 4 shows the score distribution of the two sets of unique peptides. Assuming that the decoy database comprised reversed sequences, it would be expected that none of the peptide hits found there would be real. Assuming that by chance some of the peptide sequences predicted for this set could be similar to those from the forward database, we can thus consider a false-negative error rate in order to determine a score threshold for both datasets. On this basis, for a 5% false-negative error rate in the decoy database, 1337 peptides would be above the threshold. Ranking the list of peptides, sorting by score, and taking 5% of the highest scoring peptides, the score threshold was set at 55 (shown in all panels of Figure 4 as a vertical blue line). When applying that score cut-off to the peptides obtained from the forward database, 1249 of 23,376 unique peptides (5.34%) from that database were "decoy" filtered. Translating this to the 8300 URFs used to detect the peptides, 1728 of these had at least one significant "decoy" peptide mapped onto it or was aligned with one such URF sequence. Therefore, 20.82% of the URFs can be considered more reliable than the rest.

**Figure 5:**
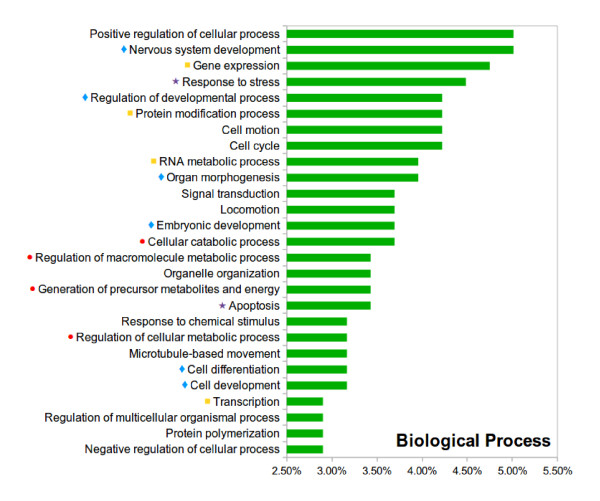
**Functional distribution of the hits based on GO annotation**. BLAST2GO multilevel ontology classification by molecular biological process over the candidate unique open reading frame sequences. Further details on the functional classes are provided in the Results section.

The sequences of all the URFs for the forward database were uploaded into the BLAST2GO software suite [22,23]. The first step was to compare those amino acid sequences to homologous proteins (using BLASTP against NCBI-nr, min e-value = 0.001, min hsp length = 25). Of the URFs with scores above decoy threshold, 1416 (81.94%) had at least a significant BLAST hit. In contrast, only 636 out of 6572 URFs with scores below the decoy threshold (10.71%) also had one or more significant BLAST hits. It was possible then to obtain a functional Gene Ontology (GO) annotation for those URFs having a BLAST hit against a known functionally annotated protein. Results of the functional annotation are summarized in Figure 5.

After GO assignment and the corresponding functional annotation of the sequences derived from our approach, enzyme codes were mapped by BLAST2GO when possible. With such codes it was possible to retrieve the KEGG pathway where the protein may play its role on the planarian molecular biology. However, less than one third of the sequences had a homologous gene/protein BLAST hit--especially for URFs dataset--, and from those many had a GO functional assignment. A fraction of the sequences with at least one GO hit was linked to an enzyme code, which would be related to a component of the KEGG pathways: 1,670 of 2,804 clusters, mapping to 118 pathways, and 131 of 5,528 clusters, mapping to 35 pathways, for MASCOT results on RefSeq and URFs respectively. All 35 pathways for URFs were also found using the RefSeq dataset. The lower ratio for the URFs set can be explained by species specific sequences, proteins or functions that are not yet annotated on the reference databases. 297 RefSeq clustered sequences had a match to 171 enzyme codes for proteins distributed on the 118 pathways. 16 URFs clustered sequences had a match to 9 enzyme codes for proteins distributed on the 35 pathways. The enzymes can appear on several pathways, due to the hierarchical structure of the KEGG a match can be found on both, a general route as "Metabolic pathway", and a more specific process, such as "Glycolysis/Gluconeogenesis". Among the pathways found, metabolism routes of sugars and lipids were expected, as energy is required for cellular processes, regeneration among them. Nevertheless, there are few candidate sequences that will deserve further analyses, as they appear on pathways close to development and regeneration: "Selenoamino acid metabolism", "Retinol metabolism in animals", and "mTOR signaling pathway". Additional data, including figures of all those pathways with color-highlighted boxes for proteins found, is available on the planarian proteomics web page [24].

### Gene profile

As depicted in Figure 5, the annotated proteins cover a wide range of biological processes, of which four main groups can be emphasized: proteins involved in energy production and metabolism (red dots in Figure 5); gene expression and transcription regulators (yellow squares); proteins related to development and differentiation (blue diamonds); and proteins involved in stress-response pathways and the apoptosis (purple stars). This functional distribution resembles the distributions described in previous studies of embryonic stem (ES) cells [25], proliferating cells [26], and differentiating neural stem cells [27], among others [28-30] (see corresponding table in Additional File 3). Additional protein sequence comparisons were performed using NCBI BLAST [31] (E-value < 10e-3) to extensively compare sets of candidate proteins from our RefSeq and URFs databases with the sequences described in those studies as stem-cell related. The same analysis was applied to the genes reported in two studies using high-throughput approaches to detect neoblast genes by RNAi-feeding [32] and by expression macrochip [33] (see corresponding table in Additional File 3). A total of 822 sequences out of 2801 (29.35%) from the RefSeq dataset and 50 out of 309 (16.18%) from the URFs dataset presented homology with at least one sequence in any of the studies. Yet only 52 (1.86%) from RefSeq and none from the URFs dataset had homology with sequences reported in the planarian studies.

### Functional studies

We performed functional analyses on some candidates from our lists to further assess the quality and accuracy of the approach used. Candidates were selected from the RefSeq and the URFs from the traces (see Table 3). In the case of RefSeq candidates, the sequence was mapped onto the draft genome and primers were designed to clone a longer fragment of the protein for subsequent characterization. Three main groups of genes were selected. The first two groups were proteins belonging to the Ras superfamily of small GTPases and the heat shock proteins (HSP) family. The third group encompassed unrelated genes from different spots. The first family includes the genes Rab-11B, Rab-39 (vesicle and membrane traffic) [34-36] and Rac-1 (cytoskeleton regulation and apoptosis) [37,38]. The second family contains HSPs (40, 60 and 70 kDa) involved in a wide variety of processes [39-41]. The last group contained the transcription factor Hunchback-like (related to *Drosophila *axial polarity and neuroblast lineage) [42], PrkC (a kinase linked to apoptosis and other processes) [43,44] and LSm proteins (RNA processing and regulation) [45-47]. This gene selection was done because no direct relation with neoblasts was established previously, with the exception of the HSPs.

**Table 3:** Summary of BLAST hits found for the analyzed candidate sequences

RefSeq candidate sequences		
**ACCESSION NUMBER**	**BLAST HOMOLOGY**	**E-VALUE**

GU591870	Rab-11B, member RAS oncogene family	1e-79

GU591871	Rab-39, Ras-related protein	1e-23

GU591872	Rac-1, ras-related	3e-90

GU591873	Hsp40 (DnaJ)	7e-18

GU591874	Hsp60	3e-103

GU591875	Hsp70 (Mortalin-like protein)	0.0

GU591876	Hunchback-like	1e-50

GU591877	PrkC (cAMP-dependent protein kinase)	2e-57

GU562964	*Smed-SmB *[58]	4e-38

**URFs candidate sequences**		

**ACCESSION NUMBER**	**BLAST HOMOLOGY**	**E-VALUE**

GU591864	Chaperonin containing TCP1 theta subunit	1e-51

GU591865	Splicing factor 3b subunit 1	6e-109

GU591866	TNF receptor associated factor	3e-25

GU591867	Similar to pol polyprotein	2e-32

GU591868	*Unknown protein*	---

GU591869	Lectin-like	4e-28

BLAST homologies to both RefSeq and URFs candidate sequences are shown. Candidate sequences coming from MASCOT predictions over the RefSeq database were mapped onto the genome draft of *Schmidtea mediterranea *to retrieve the specific sequences for this species. BLASTP was performed with the species-specific protein sequences against NCBI-nr in order to annotate their function accurately.

**Figure 6:**
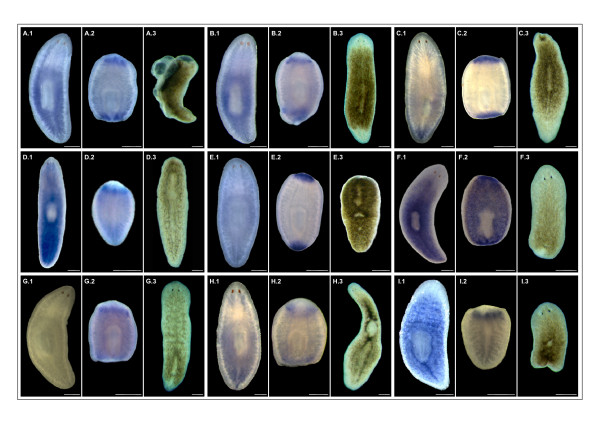
**Functional analyses of candidate genes from the RefSeq database**. Expression profiles and RNAi phenotypes are shown for a set of selected genes. A, Rab-11B; B, Rab-39; C, Rac-1; D, Hsp40; E, Hsp60; F, Hsp70; G, Hunchback-like; H, PrkC; I, *Smed-SmB*. Expression analyses by whole mount in situ hybridization in intact (A1-I1) and regenerating (A2-I2) animals are shown. In regenerating animals, the genes are expressed in the blastema and postblastema regions, areas where the neoblasts represent the main population during regeneration. In intact animals, the signal is weak for some of the genes analyzed, although the genes for which expression was detectable presented a pattern with a typical neoblast distribution. This pattern encompasses the parenchyma of the whole body excluding the gut, pharynx, and the anterior region of the head. Knock-down experiments by RNA interference were performed to further address the association of the selected genes with neoblasts (A3-I3). Detectable phenotypes were obtained in all cases except for B3 and G3. A3, E3, F3 and I3 show the phenotypes affecting the regeneration process, while C3, D3, and H3 show phenotypes affecting the intact animals. Scale bars: 250 μm.

To assess the relationship between these genes and the neoblasts, we analyzed their expression patterns and RNAi phenotypes (Figure 6). The observed expression patterns were variable. Some of the genes were expressed in the blastema (Figure 6C and 6E), where neoblasts migrate to after division in order to regenerate the missing body parts. Others were expressed in the post-blastema (Figure 6B, D, G, H and 6I), where the neoblast population is amplified by division to generate the cells that will form the blastema. Finally, some genes were expressed in both blastema and post-blastema (Figure 6A and 6F). These expression patterns disappeared in late stages of regeneration or developed over time to correspond to the typical expression pattern for neoblasts, distributed throughout the parenchyma with no expression in the pharynx or at the head tip anterior to the eyes [5]. In addition, for some of the genes, expression was only detectable under regeneration conditions, in which neoblasts are known to proliferate at higher rates. In that case, expression was barely detectable when only a basal number of neoblast cells was present in intact adult animals (Figure 6C, E and 6G). Therefore, the expression patterns for the candidate genes were consistent with neoblast expression.

Since neoblasts are known to be the only source of cells for homeostasis and regeneration, the relationship between the selected genes and the neoblasts was validated by RNAi experiments [48,49]. All injected animals, both intact and regenerating, died within a few days or weeks, except in the case of Rab39 and Hunchback-like (Figure 6B and 6G), for which no phenotype was observed in RNAi experiments. Intact planarians showed a gradual head regression followed by lysis after several weeks, as shown in Figure 6C, D and 6H. This phenotype has been linked to a lack of neoblast cells available for cell renewal [50]. In addition, regeneration was completely absent in fragments from RNAi-treated animals, which produced small blastemas that never differentiated, or no blastema at all with indented wounds, as illustrated in Figure 6A, E, F and 6I.

**Figure 7:**
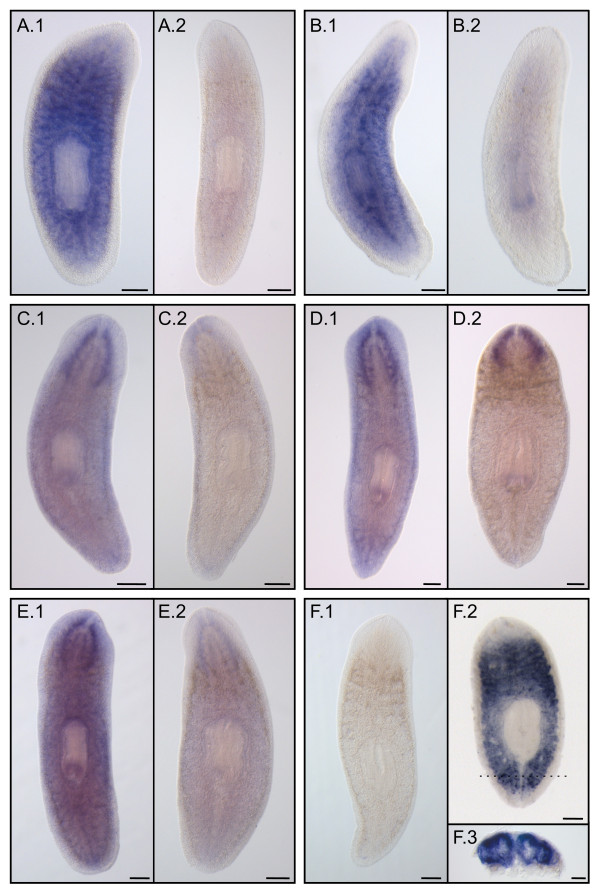
**Expression patterns of candidate genes from the *Schmidtea mediterranea *traces database**. Expression in whole mount in situ hybridization of different genes in (1) control and (2) 75 Gy irradiated planarians 6 days after irradiation. A, chaperonin containing TCP1 theta subunit homolog; B, splicing factor 3b subunit 1 homolog; C, TNF receptor-associated factor homolog; D, similar to pol polyprotein; E, unknown protein; F, lectin-like homolog. F.3 shows a higher magnification view of a transverse section from F.2 (dashed line), where the two posterior gut branches were labeled. Scale bars: 250 μm in panels A.1 to F.2 and 100 μm in panel F.3.

In a second screen to validate candidate URFs from the traces, the expression of some of these genes was analyzed by comparing intact and irradiated organisms. Whole-mount in situ hybridization in intact adult organisms revealed parenchymal expression consistent with a neoblast distribution, whereas this expression pattern was not present in irradiated animals (Figure 7A-B). This is consistent with neoblast-related genes, since high-dose irradiation destroys neoblasts. Some genes showed additional expression around the CNS that may have been associated with a non-dividing neural precursor cell type. While this expression pattern remained after irradiation, the signal in the parenchyma disappeared (Figure 7C-E). Finally, the planarian ortholog of C-type lectin-like was only expressed in the digestive system of irradiated organisms and never in intact animals (Figure 7F), suggesting a role in cell renewal under stress conditions, given that the gut has the fastest cell turnover of all tissues. These data provide further support for the involvement of these candidate genes in processes linked to neoblast biology, such as proliferation, cell migration or the regulation of differentiation.

## Discussion

The results of this study show that we have successfully developed a rapid and reliable method for 2D analysis of planarian protein samples (Figure 2 and Additional files 1 and 2). This approach will provide the basis for future proteomics studies that will increase our understanding of a number of biological processes, in planarians and beyond, building upon data obtained using genomics and cDNA-based approaches.

Proteomic studies can help to fill gaps on the annotation of the planarian genome. Despite the large number of entries already submitted, sequence databases such as NCBI [51] or UniProt [52] are far from complete. Recent metagenomic projects have identified novel putative protein sequences not present in current sequence databases, thus extending the range of biological functions that may be represented [53]. For instance, Yooseph et al [54] report up to 1 in 3 orphan ORFs from whole-genome shotgun sequencing of marine samples containing a mixture of prokaryotic organisms. Our findings indicate that MASCOT can assign substantially more peaks on those spots selected from 2D gels when using the Smed_URF database than with NCBI-nr/RefSeq, as would be expected.

The use of ORF sequences in whole genomes without prior knowledge of where the genes, mainly the exons, are located presents a number of issues that can distort the measures used to discriminate between true and false peptide hits. These include the ratio of coding to non-coding sequences, which can be quite low (around 2% of coding regions for the human genome [55]), and the presence of more repetitive sequences in intergenic regions, despite the fact that some amino acid repeats are vital functional and structural regions in proteins [56]. Moreover, the experimental spectra are compared to simulated ones that were computed from putative protein-coding regions directly translated from genomic sequences of the same species, not from related homologs from different organisms at different phylogenetic distances.

Galindo et al. [57] described a novel family of eukaryotic coding genes consisting of peptides shorter than 50 amino acids (small ORFS [smORFs]) with key biological functions during *Drosophila *development. Therefore, future searches will have to take this into account, for instance removing any length constraint when building up the ORF databases.

### Identification of proteins

Apart from the presence of metabolic proteins that indicate the high metabolic rate of neoblasts, several of the proteins detected in this analysis seem to be good candidates to be involved in neoblast-related functions, and thus in regeneration and tissue homeostasis. One of those, *Smed-SmB*, from the LSm family, has been analyzed in detail and shown to be essential for neoblast proliferation and maintenance [58]. Moreover, other candidates belonging to the HSP class of proteins have been linked to the biology of neoblasts in recent studies [59-61]. The experimental results described in this paper support the use of an ORF database built upon genomic sequences from the same species, which yields, as one might expect, more reliable results in subsequent proteomic searches, despite assuming nothing about the coding content of those ORFs. This will bridge the gap between proteomic and genomic approaches to extend our knowledge of the functional components of emerging model organisms.

### An initial proteomic picture of the neoblasts

The genes identified in this study represent the first list of neoblast-related candidate genes identified using a proteomic approach in planarias (Table 3 and Additional file 4). The results show little correspondence to those of previous genomic studies [32,33]. Interestingly, however, a number of the genes reported in this analysis were also present in studies designed to identify stem cell-specific genes in other model organisms [25-30]. In addition, five of the neoblast-related genes characterized through our proteomic approach (Hsp40, Hsp60, Hsp70, Chaperonin containing TCP1 theta subunit and Splicing factor 3b subunit 1) have also been analyzed in a planarian transcription macrochip, but only one of them was detected (Hsp60) [33]. These findings support our proteomic strategy as a complement to genomic approaches. Furthermore, the large number of putative neoblast-related proteins identified in this proteomic study will be of invaluable help in future research investigating the biology of the neoblast.

## Conclusions

We have developed a proteomic approach to characterize specific planarian stem-cell (neoblast) proteins. An accurate and reproducible method for protein purification, 2D gel electrophoresis and MS analysis was defined and an ORF database of species-specific genomic DNA was developed for peptide assignment of the retrieved MS spectra. Subsequent computational analyses yielded a list of annotated candidate proteins, some of which were functionally validated as neoblast-specific genes by RNAi and whole-mount in situ hybridization. Substantial overlap was observed between the candidate genes identified in our study and those reported from previous analyses of embryonic stem cells, thus validating the specificity of the approach. In addition, we detected novel sequence candidates and expression changes that merit further investigation in future studies to determine their role in stem-cell biology.

## Methods

### Sequences

The genome of *S. mediterranea *(strain S2F2) was sequenced and assembled at the Genome Sequencing Center (GSC) at Washington University in Saint Louis (WUSTL) [62,63]. It contains around 800 Mbp distributed on four chromosomes (2n = 8). The latest assembly version, v3.1 [21], comprises up to 90,000 sequences, which were reduced to 45,000 by means of pair-ends sequencing. Lengths of those sequences range from thousands to hundreds of thousands of nucleotides. During the assembly process, sequencing errors can be fixed by aligning different traces, but the software can also reduce polymorphisms and misplace those trace sequences because of the repeats. In order to overcome those limitations, a database of ORFs was produced directly from the set of the whole-genome shotgun reads. About 16 million traces were downloaded from the NCBI Trace Archive [64] and translated, without prior masking, into the six possible reading frames, taking into account only those ORF sequences longer than at least 50 amino acids. The ORFs were stored in a MySQL relational database along with the original sequences, to make it possible to retrieve the original nucleotide sequences and design probes for experimental validation. To reduce the large amount of sequence data produced and thus speed up the peptide searches by MASCOT [65], a set of URFs was derived from the set of ORFs with a checksum function to generate hash keys as unique identifiers for every sequence. A total of 54,382,803 ORFs were retrieved from 16,580,722 shotgun reads. This resulted in 28,946,081 URFs with properly formatted sequences to populate a MASCOT database. As MASCOT was not able to work with databases larger than 24 million entries, the original set was split into two databases. MASCOT results for both sets were then merged to get the final set of ORFs that had at least one peptide matching spectra. The probability of false matches increases when large databases, with millions of protein sequences, are used to detect a wide variety of possible candidate proteins in a sample [66,67]. To assess the significance of the peptide hits found by MASCOT, a decoy database was built by reversing all the URF sequences [68-70]. It was also split into two, as described above for the "forward" database. MASCOT was run separately on the decoy databases for all the mass fingerprints previously analysed with the original URF dataset.

### Irradiation

Intact asexual planarians were irradiated at 75 Gy (1,66 Gy/minute) with a Gammacell 1000 [Atomic Energy of Canada Limited] [71].

### Sample preparation

Protein samples were obtained from whole animals using a lysis buffer and heating. See Additional File 1 for further details.

### Running 2D gels

First-dimension isoelectric focusing was performed on immobilized pH gradient strips (24 cm, pH 3-10) using an Ettan IPGphor system. Second-dimension SDS-PAGE was performed by laying the strips on 12.5% isocratic Laemmli gels (24 × 20 cm) cast in low-fluorescence glass plates on an Ettan DALT system. Details of the procedure are available in the Additional File 1.

### Sample analysis

Gel spots were extracted and digested before analysis by MS. Then, MASCOT software (Matrix Science, London, UK) was used to search those spectra on different databases. All spectra were processed by PRIDE Converter software [72] and were submitted to the PRIDE database [73], project accession number is **15541**. For details see Additional File 1. After careful selection of score thresholds for the predicted peptides (see the Results section for the values chosen and the final numbers of the filtered datasets), the sequences that allowed detection of the URFs were uploaded into BLAST2GO [22,23]. This software tool facilitates high-throughput integration of sequence data, homology to related species via NCBI-BLAST [31] and functional annotations of DNA or protein sequences based on the Gene Ontology (GO) classification [74]. MASCOT output files, selected peptide and protein sequences, as well as BLAS2GO results and KEGG summary, are available at the planarian proteomics materials web page [24].

### Gene Cloning

Gene identifiers and corresponding forward/reverse primers (including nested primers). GU591870: F1.5'-TCTGGGATACTGCAGTCC-3', R1.5'-GATGGAATAATCGGTTGCG-3';GU591871: F1.5'-TTTTAATTGGTGATAGCATGG-3', R1.5'-CTTGACCTGCTGTATCCC-3';GU591872: F1.5'-TGTTGTTGGTGACGGAGC-3', R1.5'-GCACGAATTGCCTCATCG-3', R2.5'-TGTTCGGACAGTGATGGG-3';GU591873: F1.5'-GACTATTATTCAATATTAGG-3', R1.5'-TACCTCATATGCTTCAGCAA-3';GU591874: F1.5'-TTGCTGAAGATGTTGACGG-3', R1.5'-AGAGCGGTACCTCCTCC-3', R2.5'-ACCTCACTACTACCACCG-3';GU591875: F1.5'-GAGACAAGCTACCAAAGATGC-3', R1.5'-CATCCGTAACATCTCCAGCAAG-3';GU591876: F1.5'-AACAAATATCTGGAATGCCC-3', R1.5'-GCTTAAAATTTCCGCGGAG-3';GU591877: F1.5'-CAATATGGCTGAGGCAGC-3', R1.5'-CTGGAGTTCCACACATCG-3', R2.5'-TGGATGGGAAATTTGCTCC-3';GU562964: F1.5'-CAACACTTCAAGATGGTCG-3', R1.5'-TTGCACCAGTACCTGGCA-3';GU591864: F1.5'-CCCAGTTCTTTTCAAGGTTTAGAAG-3', F2.5'-CTGTCTTCCGAAATATCCAAGCATGC-3', R1.5'-CCAAAGATTTTGGAATTTACTGCCGTTCG-3', R2.5'-CTTTACCAACAGATTCTTCGTCACG-3';GU591865: F1.5'-GCTCATGCGCTTGGCATTCGTATTTG-3', F2.5'-CGTTTCTGAAGGCTGTGTGCAAATC-3', R1.5'-CAATGGTGTCCGCGCCTTGAGCAAC-3', R2.5'-CAATTGCTCCTCCAACCGAATGTC-3';GU591866: F1.5'-GCAACAGATGACCAACAATATAAAGG-3', F2.5'-CTAGAAACCAACAATTTTATAGCCAG-3', R1.5'-CTTGTCCGGCCTCTCTACTTC-3', R2.5'-GATTATCTTCTCGCAAGAATCCTTCTC-3';GU591867: F1.5'-CCAGCTTTCTCAACAAAGACGGGAC-3', F2.5'-GTTTCAACAGAATGCCGTTTGGAATTGC-3', R1.5'-CCGGAAAACATAAGATTGGCGCCGTC-3', R2.5'-GTTTCAAACCCTCAAACACGCTATTCG-3';GU591868: F1.5'-GCACTAGATCAAAAAATAGAAGTGTTAGC-3', F2.5'-CTCAAGAAATGGAGGAACCAAGATTGG-3', R1.5'-CGATCTACTTCTTCTACAATCTC-3', R2.5'-CTGTTTCGTCTTCTCTTGACACGTTC-3';GU591869: F1.5'-GGCTAGGTAAGTATTGGATAGATGG-3', F2.5'-GGAACTGGACGATGGGTTGATAG-3', R1.5'-CCAATTTGTGTAGGTCATTTTGCATCC-3', R2.5'-CCATCATTGAATGTCCATCTTCCAGTG-3'.

### In situ hybridization

Digoxigenin-labeled RNA probes were prepared using an in vitro labeling kit (Roche). Whole-mount in situ hybridization was performed as described by Agata et al [75], with some modifications: proteinase K (20 μg/ml) treatment for 10 min; triethanolamine treatment was performed as described by Nogi and Levin [76]; hybridization at 55°C for 18 or 30 h; and final probe concentration of 0.07 ng/μl.

### RNA interference

Double-stranded RNAs (dsRNA) were produced by *in vitro *transcription (Roche) and injected into the gut of the planarians as described in Sánchez-Alvarado and Newmark [49]. Three aliquots of 32 nl (400-800 ng/μl) were injected on three consecutive days with a Drummond Scientific Nanoject injector (Broomall, PA). On the fourth or fifth day, some of the planarians were amputated while the rest were left intact. Control organisms were injected with water.

## Abbreviations

EST: expressed sequence tags; MS: mass spectrometry; CB: chromatoid bodies; 2D gel: two-dimensional gel; DIGE: difference in gel electrophoresis; cm: centimeters; Ip: Isoelectric point; MW: Molecular weight; WT: wild type; IA: irradiated animals; H3P: phosphorylated histone H3; ORF: open reading frame; URF: unique ORF; NCBI-nr: NCBI non-redundant (database); WUSTL: Washington University in Saint Louis; hsp: high-scoring segment pair (BLAST); GO: Gene Ontology; EC: Enzyme Code (KEGG); ES: embryonic stem cells; HSP/Hsp: heat shock protein; kDa: kilodalton; RNAi: RNA interference; CNS: central nervous system; Gy: grays; dsRNA: double-stranded RNA.

## Authors' contributions

EFT, ES and JFA conceived of the study. EFT ran the 2D gels and counted the spots. JFA performed the computational analyses, compiled the sequence databases, processed the MASCOT results, and ran the GO functional and KEGG annotation. EFT ran the MASCOT searches and produced the initial BLAST annotation for RefSeq candidates. EFT and GRE performed the experimental validation of the selected protein candidates. All authors participated in its design and coordination, helped to draft the manuscript, and read and approved the final manuscript.

## Supplementary Material

Additional file 1 **Details on Material and Methods**. An extended description of the proteomics protocols applied to perform the analyses presented on this paper. Click here for file

Additional file 2 **Image scans of all silver-stained 2D gel replicates**. Image scans of different and independent silver-stained 2D gels used in the study. A to D and the respective zooms, for the regions delimited by red squares, I to L, come from 100 μg of loaded samples. E to H and the respective zooms M to P correspond to 500 μg loaded samples. A, C, E and G are control samples. B, D, F and H are irradiated samples. Although the staining and running conditions were not exactly equivalent, one can observe that the spot pattern shown by all the gels is repetitive, which is more evident on the zoomed regions. Click here for file

Additional file 3 **Comparing the results presented in this manuscript with previously published studies relating to stem cells**. Comparison of candidate neoblast protein sequences presented in this paper with genes reported in other proteomic studied to be related to stem cells [25-30] and with specific neoblast-related genes identified in two different high-throughput approaches [32,33]. From the URFs database, only those sequences with a positive decoy were selected. NCBI BLASTP [31] (min e-value = 0.001) was used on sequence comparison. Sequences were clustered according to their homology and they are listed in the table by their original GI identifier from the corresponding NCBI database. Click here for file

Additional file 4 **Table of peptide candidates**. Listing of the sequence candidates obtained from the computational analysis of the raw proteomics data over the RefSeq and URF datasets (see the corresponding sheet on the spreadsheet file). Only those with a significant BLAST hit are shown (using BLASTP against NCBI-nr, min e-value = 0.001, min hsp length = 25). Genes described in detail in Table 3 are not included. The sequences in this table were built from sets of URFs derived from traces; we provide the corresponding trace identifiers from Genbank TraceDB [64]. Click here for file

## References

[B1] Beyer A Hollunder J Nasheuer HP Wilhelm T Post-transcriptional expression regulation in the yeast *Saccharomyces cerevisiae *on a genomic scale Mol Cell Proteomics 2004 3 11 1083 1092 10.1074/mcp.M400099-MCP200 15326222

[B2] Pandey A Mann M Proteomics to study genes and genomes Nature 2000 405 6788 837 846 10.1038/35015709 10866210

[B3] Hanash S Disease proteomics Nature 2003 422 6928 226 232 10.1038/nature01514 12634796

[B4] Khan SM Franke-Fayard B Mair GR Lasonder E Janse CJ Mann M Waters AP Proteome analysis of separated male and female gametocytes reveals novel sex-specific *Plasmodium *biology Cell 2005 121 5 675 687 10.1016/j.cell.2005.03.027 15935755

[B5] Handberg-Thorsager M Fernández-Taboada E Saló E Stem cells and regeneration in planarians Front Biosci 2008 13 6374 6394 10.2741/3160 18508666

[B6] Saló E The power of regeneration and the stem-cell kingdom: freshwater planarians (Platyhelminthes) Bioessays 2006 28 5 546 559 16615086 10.1002/bies.20416

[B7] Sánchez-Alvarado A Newmark PA Robb SM Juste R The *Schmidtea mediterranea *database as a molecular resource for studying platyhelminthes, stem cells and regeneration Development 2002 129 24 5659 5665 12421706 10.1242/dev.00167

[B8] Baguñà J Saló E Auladell C Regeneration and pattern formation in planarians III. Evidence that neoblasts are totipotent stem cells and the source of blastema cells Development 1989 107 77 86

[B9] Newmark PA Sánchez-Alvarado A Bromodeoxyuridine specifically labels the regenerative stem cells of planarians Dev Biol 2000 220 2 142 153 10.1006/dbio.2000.9645 10753506

[B10] Coward SJ Chromatoid bodies in somatic cells of the planarian: observations on their behavior during mitosis Anat Rec 1974 180 3 533 545 10.1002/ar.1091800312 4371241

[B11] Gremigni V Planarian regeneration: An overview of some cellular mechanisms Zool Sci 1988 5 1153 1163

[B12] Higuchi S Hayashi T Hori I Shibata N Sakamoto H Agata K Characterization and categorization of fluorescence activated cell sorted planarian stem cells by ultrastructural analysis Dev Growth Differ 2007 49 7 571 581 10.1111/j.1440-169X.2007.00947.x 17587325

[B13] Wolff E Dubois F Sur la migration des cellules de régénération chez les planaires Rev Suisse Zool 1948 55 218 227

[B14] Bayascas JR Castillo E Muñoz-Mármol AM Saló E Planarian Hox genes: novel patterns of expression during regeneration Development 1997 124 1 141 148 9006075 10.1242/dev.124.1.141

[B15] Collet J Baguñà J Optimizing a method of protein extraction for two-dimensional electrophoretic separation of proteins from planarians (Platyhelminthes, Turbellaria) Electrophoresis 1993 14 10 1054 1059 10.1002/elps.11501401168 8125055

[B16] Garbis S Lubec G Fountoulakis M Limitations of current proteomics technologies Journal of Chromatography A 2005 1077 1 1 18 10.1016/j.chroma.2005.04.059 15988981

[B17] MASCOT search engine to identify proteins from primary sequence databases using mass spectrometry data http://www.matrixscience.com/

[B18] Sayers EW Barrett T Benson DA Bryant SH Canese K Chetvernin V Church DM DiCuccio M Edgar R Federhen S Database resources of the National Center for Biotechnology Information Nucleic Acids Res 2009 37 Database D5 15 10.1093/nar/gkn741 PMC2686545 18940862

[B19] Pruitt Kim D Tatusova T Maglott DR NCBI reference sequences (RefSeq): a curated non-redundant sequence database of genomes, transcripts and proteins Nucleic Acids Res 2007 35 Database D61 D65 10.1093/nar/gkl842 17130148 PMC1716718

[B20] Zayas RM Hernandez A Habermann B Wang Y Stary JM Newmark PA The planarian *Schmidtea mediterranea *as a model for epigenetic germ cell specification: analysis of ESTs from the hermaphroditic strain Proc Natl Acad Sci USA 2005 102 51 18491 18496 10.1073/pnas.0509507102 16344473 PMC1317986

[B21] Robb SM Ross E Sánchez-Alvarado A SmedGD: the Schmidtea mediterranea genome database Nucleic Acids Res 2008 36 Database D599 606 17881371 10.1093/nar/gkm684 PMC2238899

[B22] Conesa A Gotz S Garcia-Gomez JM Terol J Talon M Robles M Blast2GO: a universal tool for annotation, visualization and analysis in functional genomics research Bioinformatics 2005 21 18 3674 3676 10.1093/bioinformatics/bti610 16081474

[B23] Gotz S Garcia-Gomez JM Terol J Williams TD Nagaraj SH Nueda MJ Robles M Talon M Dopazo J Conesa A High-throughput functional annotation and data mining with the Blast2GO suite Nucleic Acids Res 2008 36 10 3420 3435 10.1093/nar/gkn176 18445632 PMC2425479

[B24] Planarian neoblast proteomics online supplementary data http://compgen.bio.ub.es/tiki-index.php?page=Planarian+Proteomics

[B25] Baharvand H Fathi A Gourabi H Mollamohammadi S Salekdeh GH Identification of mouse embryonic stem cell-associated proteins J Proteome Res 2008 7 1 412 423 10.1021/pr700560t 18047272

[B26] Hoffrogge R Mikkat S Scharf C Beyer S Christoph H Pahnke J Mix E Berth M Uhrmacher A Zubrzycki IZ 2-DE proteome analysis of a proliferating and differentiating human neuronal stem cell line (ReNcell VM) Proteomics 2006 6 6 1833 1847 10.1002/pmic.200500556 16475233

[B27] Maurer MH Feldmann RE Jr Futterer CD Butlin J Kuschinsky W Comprehensive proteome expression profiling of undifferentiated versus differentiated neural stem cells from adult rat hippocampus Neurochem Res 2004 29 6 1129 1144 10.1023/B:NERE.0000023600.25994.11 15176470

[B28] Kohler C Wolff S Albrecht D Fuchs S Becher D Buttner K Engelmann S Hecker M Proteome analyses of *Staphylococcus aureus *in growing and non-growing cells: a physiological approach Int J Med Microbiol 2005 295 8 547 565 10.1016/j.ijmm.2005.08.002 16325551

[B29] Nagano K Taoka M Yamauchi Y Itagaki C Shinkawa T Nunomura K Okamura N Takahashi N Izumi T Isobe T Large-scale identification of proteins expressed in mouse embryonic stem cells Proteomics 2005 5 5 1346 1361 10.1002/pmic.200400990 15742316

[B30] Zenzmaier C Kollroser M Gesslbauer B Jandrositz A Preisegger KH Kungl AJ Preliminary 2-D chromatographic investigation of the human stem cell proteome Biochem Biophys Res Commun 2003 310 2 483 490 10.1016/j.bbrc.2003.09.036 14521936

[B31] Altschul SF Gish W Miller W Myers EW Lipman DJ Basic local alignment search tool J Mol Biol 1990 215 3 403 410 2231712 10.1016/S0022-2836(05)80360-2

[B32] Reddien PW Bermange AL Murfitt KJ Jennings JR Sánchez-Alvarado A Identification of genes needed for regeneration, stem cell function, and tissue homeostasis by systematic gene perturbation in planaria Dev Cell 2005 8 5 635 649 10.1016/j.devcel.2005.02.014 15866156 PMC2267917

[B33] Rossi L Salvetti A Marincola FM Lena A Deri P Mannini L Batistoni R Wang E Gremigni V Deciphering the molecular machinery of stem cells: a look at the neoblast gene expression profile Genome Biol 2007 8 4 R62 10.1186/gb-2007-8-4-r62 17445279 PMC1896013

[B34] Stenmark H Olkkonen VM The Rab GTPase family Genome Biol 2001 2 5 REVIEWS3007 10.1186/gb-2001-2-5-reviews3007 11387043 PMC138937

[B35] Segev N Ypt and Rab GTPases: insight into functions through novel interactions Curr Opin Cell Biol 2001 13 4 500 511 10.1016/S0955-0674(00)00242-8 11454458

[B36] Cheng H Ma Y Ni X Jiang M Guo L Ying K Xie Y Mao Y Isolation and characterization of a human novel RAB (RAB39B) gene Cytogenet Genome Res 2002 97 1-2 72 75 10.1159/000064047 12438742

[B37] Aznar S Lacal JC Rho signals to cell growth and apoptosis Cancer Lett 2001 165 1 1 10 10.1016/S0304-3835(01)00412-8 11248412

[B38] Raftopoulou M Hall A Cell migration: Rho GTPases lead the way Dev Biol 2004 265 1 23 32 10.1016/j.ydbio.2003.06.003 14697350

[B39] Beere HM Green DR Stress management-heat shock protein-70 and the regulation of apoptosis Trends Cell Biol 2001 11 1 6 10 10.1016/S0962-8924(00)01874-2 11146277

[B40] Hartl FU Hayer-Hartl M Molecular chaperones in the cytosol: from nascent chain to folded protein Science 2002 295 5561 1852 1858 10.1126/science.1068408 11884745

[B41] Rutherford SL Lindquist S Hsp90 as a capacitor for morphological evolution Nature 1998 396 6709 336 342 10.1038/24550 9845070

[B42] Pearson BJ Doe CQ Regulation of neuroblast competence in Drosophila Nature 2003 425 6958 624 628 10.1038/nature01910 14534589

[B43] Abdel-Raheem IT Hide I Yanase Y Shigemoto-Mogami Y Sakai N Shirai Y Saito N Hamada FM El-Mahdy NA Elsisy Ael D Protein kinase C-alpha mediates TNF release process in RBL-2H3 mast cells Br J Pharmacol 2005 145 4 415 423 10.1038/sj.bjp.0706207 15806111 PMC1576159

[B44] Nakajima T Signaling cascades in radiation-induced apoptosis: roles of protein kinase C in the apoptosis regulation Med Sci Monit 2006 12 10 RA220 224 17006414

[B45] Beggs JD Lsm proteins and RNA processing Biochem Soc Trans 2005 33 Pt 3 433 438 15916535 10.1042/BST0330433

[B46] He W Parker R Functions of Lsm proteins in mRNA degradation and splicing Curr Opin Cell Biol 2000 12 3 346 350 10.1016/S0955-0674(00)00098-3 10801455

[B47] Tharun S He W Mayes AE Lennertz P Beggs JD Parker R Yeast Sm-like proteins function in mRNA decapping and decay Nature 2000 404 6777 515 518 10.1038/35006676 10761922

[B48] Pineda D Gonzalez J Callaerts P Ikeo K Gehring WJ Saló E Searching for the prototypic eye genetic network sine oculis is essential for eye regeneration in planarians Proc Natl Acad Sci USA 2000 97 9 4525 4529 10.1073/pnas.97.9.4525 10781056 PMC18268

[B49] Sánchez Alvarado A Newmark PA Double-stranded RNA specifically disrupts gene expression during planarian regeneration Proc Natl Acad Sci USA 1999 96 9 5049 5054 10220416 10.1073/pnas.96.9.5049 PMC21814

[B50] Salvetti A Rossi L Deri P Batistoni R An MCM2-related gene is expressed in proliferating cells of intact and regenerating planarians Developmental Dynamics 2000 218 4 603 614 10.1002/1097-0177(2000)9999:9999<::AID-DVDY1016>3.0.CO;2-C 10906779

[B51] Benson DA Karsch-Mizrachi I Lipman DJ Ostell J Wheeler DL GenBank Nucleic Acids Res 2008 36 Database D25 30 18073190 10.1093/nar/gkm929 PMC2238942

[B52] The UniProt Consortium The universal protein resource (UniProt) Nucleic Acids Res 2008 36 Database D190 195 18045787 10.1093/nar/gkm895 PMC2238893

[B53] Pignatelli M Aparicio G Blanquer I Hernandez V Moya A Tamames J Metagenomics reveals our incomplete knowledge of global diversity Bioinformatics 2008 1524 18 2124 5 10.1093/bioinformatics/btn355 PMC2530889 18625611

[B54] Yooseph S Sutton G Rusch DB Halpern AL Williamson SJ Remington K Eisen JA Heidelberg KB Manning G Li W The Sorcerer II Global Ocean Sampling expedition: expanding the universe of protein families PLoS Biol 2007 5 3 e16. 10.1371/journal.pbio.0050016 17355171 PMC1821046

[B55] International Human Genome Sequencing Consortium Finishing the euchromatic sequence of the human genome Nature 2004 431 7011 931 945 10.1038/nature03001 15496913

[B56] Kalita MK Ramasamy G Duraisamy S Chauhan VS Gupta D ProtRepeatsDB: a database of amino acid repeats in genomes BMC Bioinformatics 2006 7 336 10.1186/1471-2105-7-336 16827924 PMC1538635

[B57] Galindo MI Pueyo JI Fouix S Bishop SA Couso JP Peptides encoded by short ORFs control development and define a new eukaryotic gene family PLoS Biol 2007 5 5 e106 10.1371/journal.pbio.0050106 17439302 PMC1852585

[B58] Fernández-Taboada E Moritz S Stehling M Zeuschner D HR S Saló E Gentile L Smed-SmB, a member of the (L)Sm protein superfamily, is essential for chromatoid body organization and planarian stem cell proliferation Development 2010 137 9 1583 10.1242/dev.042564 20215344

[B59] Conte M Deri P Isolani ME Mannini L Batistoni R A mortalin-like gene is crucial for planarian stem cell viability Dev Biol 2009 334 1 109 118 10.1016/j.ydbio.2009.07.010 19616535

[B60] Conte M Isolani ME Deri P Mannini L Batistoni R Expression of hsp90 mediates cytoprotective effects in the gastrodermis of planarians Cell Stress Chaperones 2011 16 1 33 39 10.1007/s12192-010-0218-6 20706815 PMC3024083

[B61] Sánchez Navarro B Michiels N Köhler H-R D'Souza T Differential expression of heat shock protein 70 in relation to stress type in the flatworm *Schmidtea polychroa* Hydrobiologia 2009 636 393 400

[B62] *Schmidtea mediterranea *genome sequencing project http://genome.wustl.edu/genomes/view/schmidtea_mediterranea/

[B63] Belleville S Beauchemin M Tremblay M Noiseux N Savard P Homeobox-containing genes in the newt are organized in clusters similar to other vertebrates Gene 1992 114 179 186 10.1016/0378-1119(92)90572-7 1351019

[B64] *Schmidtea mediterranea *trace archive at NCBI ftp://ftp.ncbi.nih.gov/pub/TraceDB/schmidtea_mediterranea/

[B65] Perkins DN Pappin DJ Creasy DM Cottrell JS Probability-based protein identification by searching sequence databases using mass spectrometry data Electrophoresis 1999 20 18 3551 3567 10.1002/(SICI)1522-2683(19991201)20:18<3551::AID-ELPS3551>3.0.CO;2-2 10612281

[B66] Cargile BJ Talley DL Stephenson JL Jr Immobilized pH gradients as a first dimension in shotgun proteomics and analysis of the accuracy of pI predictability of peptides Electrophoresis 2004 25 6 936 945 10.1002/elps.200305722 15004858

[B67] Resing KA Meyer-Arendt K Mendoza AM Aveline-Wolf LD Jonscher KR Pierce KG Old WM Cheung HT Russell S Wattawa JL Improving reproducibility and sensitivity in identifying human proteins by shotgun proteomics Anal Chem 2004 76 13 3556 3568 10.1021/ac035229m 15228325

[B68] Elias JE Gygi SP Target-decoy search strategy for increased confidence in large-scale protein identifications by mass spectrometry Nat Methods 2007 4 3 207 214 10.1038/nmeth1019 17327847

[B69] Higdon R Hogan JM Kolker N van Belle G Kolker E Experiment-specific estimation of peptide identification probabilities using a randomized database Omics 2007 11 4 351 365 10.1089/omi.2007.0040 18092908

[B70] Higdon R Hogan JM Van Belle G Kolker E Randomized sequence databases for tandem mass spectrometry peptide and protein identification Omics 2005 9 4 364 379 10.1089/omi.2005.9.364 16402894

[B71] Saló E Baguñà J Cell movement in intact and regenerating planarians. Quantitation using chromosomal, nuclear and cytoplasmic markers J Embryol Exp Morphol 1985 89 57 70 3867725

[B72] Barsnes H Vizcaino JA Eidhammer I Martens L PRIDE Converter: making proteomics data-sharing easy Nat Biotechnol 2009 27 7 598 599 10.1038/nbt0709-598 19587657

[B73] Vizcaino JA Cote R Reisinger F Foster JM Mueller M Rameseder J Hermjakob H Martens L A guide to the Proteomics Identifications Database proteomics data repository Proteomics 2009 9 18 4276 4283 10.1002/pmic.200900402 19662629 PMC2970915

[B74] Ashburner M Ball CA Blake JA Botstein D Butler H Cherry JM Davis AP Dolinski K Dwight SS Eppig JT Gene ontology: tool for the unification of biology. The Gene Ontology Consortium Nat Genet 2000 25 1 25 29 10.1038/75556 10802651 PMC3037419

[B75] Agata K Soejima Y Kato K Kobayashi C Umesono Y Watanabe K Structure of the planarian central nervous system (CNS) revealed by neuronal cell markers Zool Sci 1998 15 433 440 10.2108/zsj.15.433 18466009

[B76] Nogi T Levin M Characterization of innexin gene expression and functional roles of gap-junctional communication in planarian regeneration Dev Biol 2005 287 2 314 335 10.1016/j.ydbio.2005.09.002 16243308

