# Supplementary material for: A proteomics approach to decipher the molecular nature of planarian stem cells
Source: BMC Genomics. 2011 Feb 28;12:133. doi: 10.1186/1471-2164-12-133 (PMC3058083; doi:10.1186/1471-2164-12-133)
Supplement: Additional file 1 — Details on Material and Methods. An extended description of the proteomics protocols applied to perform the analyses presented on this paper. [file 1471-2164-12-133-S1.DOC]

# DETAILS on MATERIAL and METHODS

**1.- Material and methods:**

**1.1.- Sample preparation for two-dimensional gel electrophoresis proteomic analysis**

Samples for proteomic analysis were prepared from a whole planarian extract with lysis buffer (2% sodium dodecyl sulfate [SDS], 40 mM TrisHCl pH 8.5, 60 mM dithiothreitol [DTT]) by heating and vortexing. The samples were further purified using a modified acetone precipitation procedure (2D Clean-up kit, GE Healthacare). Finally, protein pellets were resuspended in differential in gel electrophoresis (DIGE) labeling buffer, and protein concentration was determined using the Bio-Rad RCDC Protein Assay (Bio-Rad, Hemel Hempstead, UK).

**1.2.- Two-dimensional gel electrophoresis**

**1.2.1.- First dimension**

First-dimension isoelectric focusing was performed on immobilized pH gradient strips (24 cm, pH 3–10) using an Ettan-dalt IPGphor System. Samples were loaded and equilibrated in a two-step incubation lasting 10-15 min, the first step with SDS-equilibration buffer (50mM Tris-HCl pH 8.8, 6M urea, 30% Glicerol, 2% SDS, bromophenol blue) + 1% DTT and the second step with SDS-equilibration buffer + 4.5% iodoacetamide. The following program was run:

- - First-dimension running program:
    1. Step and hold.........50V.........12h (Rehydration)
    2. Step and hold........500V..........1h
    3. Step and hold......1000V..........1h
    4. Gradient..............8000V..........3h
    5. Step and hold......8000V...40000 v·h
    6. Step and hold........100V.........10h

**1.2.2.- Second dimension**

Second-dimension SDS polyacrylamide gel electrophoresis (PAGE) was performed by equilibrating the strip 30 min in equilibration buffer and then laying the strips on acrylamide gels (24×20 cm), cast in glass plates, on an Amersham system. Gels were run at 20ºC at a constant power of 15 mA/gel until the sample came in to the acrylamide gel from the first dimension strip followed by 30 mA/gel until the bromophenol blue tracking front had run off the end of the gel. Once completed, the gels were fixed overnight in 10% AcOH and 30% EtOH.

**1.3.- Silver staining**

Silver staining was performed using a modified Blum’s protocol, adapted from Mortz et al. [1]. The gels were fixed 1 h (or overnight) in 30% CH_3_CH_2_OH and 10% CH_3_COOH. Then, the gels were washed three times for 20 min in 30% CH_3_CH_2_OH (5 x 20 min if overnight fixation was used) followed by a sensitization step of 1 min in 0.02% Na_2_S_2_O_3_. Three rapid washes of 30 sec were performed after the sensitization and the gels were stained with 0.2% AgNO_3_ for 20 min. Three H_2_O washes of 30 sec were done and then developed with 6% Na_2_CO_3_, 0.0004% Na_2_S_2_O_3_, and 0.05% HCHO (added just prior to use). Once the signal was correct we washed three times for 30 min with H_2_O and finally stopped the development with 30% CH_3_CH_2_OH and 10% CH_3_COOH 10 min (or overnight).

**1.4.- In-gel digestion**

Proteins were in-gel digested with trypsin (sequencing grade modified, Promega) in an automatic Investigator ProGest robot (Genomic Solutions). Briefly, excised gel spots were washed sequentially with ammonium bicarbonate buffer (50 mM NH_4_HCO_3_) and acetonitrile. Proteins were reduced and alkylated by treatment with 10 mM DTT solution for 30 min and treatment with a 55 mM solution of iodine acetamide, respectively. After sequential washes with buffer and acetronitrile, proteins were digested overnight at 37ºC with 0.27 nmol of trypsin. Tryptic peptides were extracted from the gel matrix with 10% formic acid and acetonitrile; the extracts were pooled and dried in a vacuum centrifuge.

**1.5.- Acquisition of MS and MS/MS Spectra.**

Proteins excised from the two-dimensional gels were analyzed in either a MALDI-TOF/TOF (4700 Proteomics Analyzer, Applied Biosystems) or LC-ESI-QTOF (Q-TOF Global, Micromass-Waters) mass spectrometer.

**1.5.1.- For MALDI-MS** analysis, the digests were redissolved in 5 μL of 0.1% trifluoroacetic acid in 50% acetonitrile. Typically a 0.5 to 1 μL aliquot was mixed with the same volume of a matrix solution, 2 to 5 mg/mL α-cyano-4-hydroxycinnamic acid (Waters) in 0.1% trifluoroacetic acid in 50% acetonitrile and spotted to the MALDI plate. Mass spectra were acquired in positive reflector mode (voltage of 20 kV in the source 1 and laser intensity ranged from 5800 to 6200). Typically, 500 shots per spectrum were accumulated. Three major peaks were selected to be further characterized by MS/MS analysis. MS/MS spectra were acquired using collision-induced dissociation (CID) with atmospheric air as the collision gas. An MS-MS 1 kV positive mode was used. MS and MS/MS spectra from the same spot were merged in a single mgf file prior to submission for database searching.

**1.5.2.- For LC-MS** analysis by on-line liquid chromatography tandem mass spectrometry (Cap-LC-nano-ESI-Q-TOF) (CapLC, Micromass-Waters), the tryptic peptide samples were resuspended in 15 μL of 1% formic acid solution and 4 μL was injected onto a reverse-phase capillary C_18_ column (75 μm of internal diameter and 15 cm length, PepMap column, LC Packings) for chromatographic separation. The eluted peptides were ionized via coated nano-ES needles (PicoTip^TM^, New Objective). A capillary voltage of 2000 to 2800 V was applied together with a cone voltage of 80 V. The collision in the CID was 20-35 eV and argon was employed as collision gas. Data were generated in PKL file format and submitted for database searching in MASCOT server.

**1.6.- Database search**

PKL and mgf files were submitted for database searching in a MASCOT search engine against different databases. The search parameters were as follows: 1 missed cleavage, fixed, and variable modifications were carbamidomethylation of cysteine and oxidation of methionine, respectively. Peptide tolerance was 100 ppm and 0.25 Da, respectively, for MS and MS/MS spectra.

**1.7.- DIGE**

DIGE protein concentration was adjusted to 2 mg/ml by the addition of the DIGE labeling buffer (7 mol/l urea, 2 mol/l thiourea, 4% w/v 3-[(3-cholamidopropyl) dimethylammonio]-1-propanesulfonate [CHAPS], 30 mmol/l Tris, pH 8.0). Labeling was carried out by the addition of 400 pmol of the required Cy dye in 1 μl of anhydrous N,N-dimethylformamide per 50 μg of protein. After 30 min of incubation on ice in the dark, the reaction was quenched with 10 mmol/l lysine and the samples incubated for a further 10 min. Samples were combined according to the experimental design, using 50 μg of protein per Cy dye per gel, and diluted two-fold with isoelectric focusing sample buffer (7 mol/l urea, 2 mol/l thiourea, 4% w/v CHAPS, 2% DTT, 2% pharmalytes, pH 3–10). One C and one Pi sample were then separated by two-dimensional electrophoresis in each of the gels.

The electrophoresis was performed using GE Healthcare reagents and equipment. First-dimension isoelectric focusing was performed on immobilized pH gradient strips (24 cm, pH 3–10) using an Ettan IPGphor System. Samples were applied near the basic end of the strips by cup-loading after being incubated overnight in 450 μl of rehydration buffer (7 mol/l urea, 2 mol/l thiourea, 4% w/v CHAPS, 1 % pharmalytes, pH 3–10, 100 mmol/l DeStreak). After focusing at 67 kV×h, strips were equilibrated, first for 15 min in 6 ml of reducing solution (6 mol/l urea, 100 mmol/l Tris-HCl, pH 8, 30% v/v glycerol, 2% w/v SDS, 5 mg/ml DTT) and then in 6 ml of alkylating solution (6 mol/l urea, 100 mmol/l Tris-HCl, pH 8, 30% v/v glycerol, 2% w/v SDS, 22.5 mg/ml iodoacetamide) for 15 min on a rocking platform. Second-dimension SDS-PAGE was performed by laying the strips on 12.5% isocratic Laemmli gels (24×20 cm), cast in low-fluorescence glass plates, on an Ettan DALT VI system. Gels were run at 20ºC at a constant power of 2.5 W per gel for 30 min followed by 17 W per gel until the bromophenol blue tracking front had run off the end of the gel.

Fluorescence images of the gels were obtained on a Typhoon 9400 scanner (GE Healthcare). Cy2, Cy3 and Cy5 images were scanned at excitation/emission wavelengths of 488/520 nm, 532/580 nm and 633/670 nm, respectively, at a resolution of 100 μm. Both image analysis and statistical quantification of relative protein levels were performed using DeCyder v. 5.0 software (GE Healthcare).

**1.8.- Protein identification by mass spectrometry**

Protein spots of interest were excised from the gel using an automated spot picker (GE Healthcare). In-gel trypsin digestion was performed as described previously [2], using autolysis-stabilized trypsin (Promega, Madison, WI, USA). Tryptic digests were purified using ZipTip microtitre plates (Millipore, Billerica, MA, USA). Peptide mass fingerprint by matrix-assisted laser desorption ionization-mass spectrometry (MALDI-MS) was used for protein identification. MALDI-MS analysis of tryptic peptides was performed according to Monge et al*.* [3]. The Mascot 2.0 program (Matrix Science, London, UK) was used to search the Matrix Science database (MSDB), 20050929 release (Imperial College, London). Search parameters were performed according to Monge et al*.* [3]. Criteria for positive identification were a significant Mascot probability score (p<0.05).

**2.- REFERENCES:**

1. Mortz E, Krogh TN, Vorum H and Gorg A. **Improved silver staining protocols for high sensitivity protein identification using matrix-assisted laser desorption/ionization-time of flight analysis.** *Proteomics* 2001, **1**:1359-63.

2. Shevchenko A, Wilm M, Vorm O, Mann M. **Mass spectrometric sequencing of proteins from silver-stained polyacrylamide gels.** *Anal Chem* 1996, **68**:850–85.

3. Monge M, Doll A, Colas E, Gil-Moreno A, Castellvi J, Garcia A, Colome N, Perez-Benavente A, Pedrola N, Lopez-Lopez R, Dolcet X, Ramon y Cajal S, Xercavins J, Matias-Guiu X, Canals F, Reventos J, Abal M. **Subtractive proteomic approach to the endometrial carcinoma invasion front.** *J Proteome Res* 2009, **8**(10):4676-84.
