# Supplementary figures and images for: A proteomics approach to decipher the molecular nature of planarian stem cells
Source: BMC Genomics. 2011 Feb 28;12:133. doi: 10.1186/1471-2164-12-133 (PMC3058083; doi:10.1186/1471-2164-12-133)

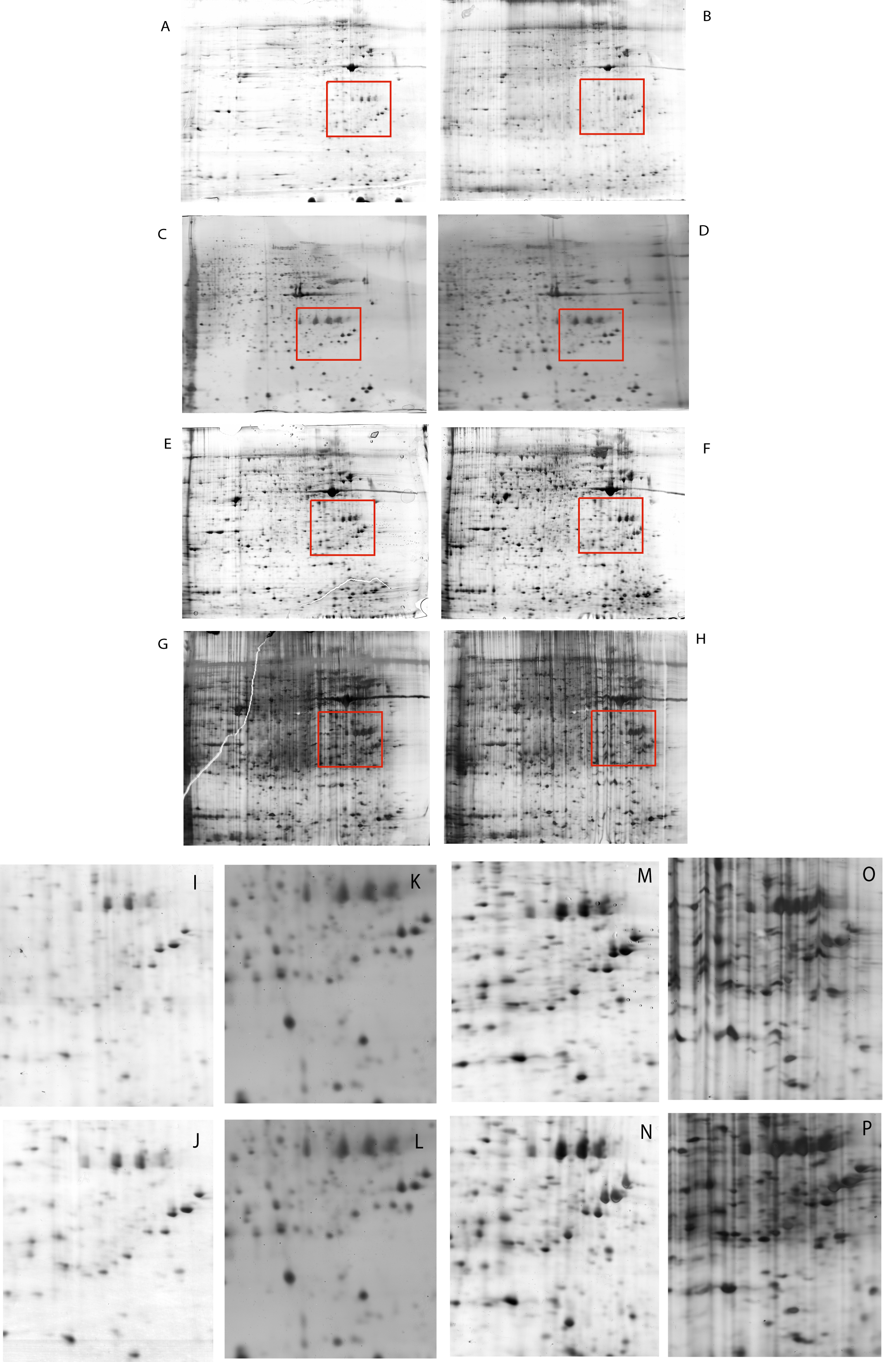

Supplement: Additional file 2 — Image scans of all silver-stained 2D gel replicates. Image scans of different and independent silver-stained 2D gels used in the study. A to D and the respective zooms, for the regions delimited by red squares, I to L, come from 100 μg of loaded samples. E to H and the respective zooms M to P correspond to 500 μg loaded samples. A, C, E and G are control samples. B, D, F and H are irradiated samples. Although the staining and running conditions were not exactly equivalent, one can observe that the spot pattern shown by all the gels is repetitive, which is more evident on the zoomed regions. [file 1471-2164-12-133-S2.TIFF]
